# Fear Recognition for Women Using a Reduced Set of Physiological Signals

**DOI:** 10.3390/s21051587

**Published:** 2021-02-25

**Authors:** Jose A. Miranda, Manuel F. Canabal, Laura Gutiérrez-Martín, Jose M. Lanza-Gutierrez, Marta Portela-García, Celia López-Ongil

**Affiliations:** 1 Electronic Technology Department, Universidad Carlos III of Madrid, 28911 Leganés, Madrid, Spain; mcanabal@ing.uc3m.es (M.F.C.); lagutier@ing.uc3m.es (L.G.-M.); marta.portela@uc3m.es (M.P.-G.); celia@ing.uc3m.es (C.L.-O.); 2 Department of Computer Science, University of Alcalá, 28871 Alcalá de Henares, Madrid, Spain; jm.lanza@uah.es

**Keywords:** fear recognition, physiological signals, signal processing, wearable sensors

## Abstract

Emotion recognition is benefitting from the latest research into physiological monitoring and wireless communications, among other remarkable achievements. These technologies can indeed provide solutions to protect vulnerable people in scenarios such as personal assaults, the abuse of children or the elderly, gender violence or sexual aggression. Cyberphysical systems using smart sensors, artificial intelligence and wearable and inconspicuous devices can serve as bodyguards to detect these risky situations (through fear-related emotion detection) and automatically trigger a protection protocol. As expected, these systems should be trained and customized for each user to ensure the best possible performance, which undoubtedly requires a gender perspective. This paper presents a specialized fear recognition system for women based on a reduced set of physiological signals. The architecture proposed is characterized by the usage of three physiological sensors, lightweight binary classification and the conjunction of linear (temporal and frequency) and non-linear features. Moreover, a binary fear mapping strategy between dimensional and discrete emotional information based on emotional self-report data is implemented to avoid emotional bias. The architecture is evaluated using a public multi-modal physiological dataset with two approaches (subject-dependent and subject-independent models) focusing on the female participants. As a result, the proposal outperforms the state-of-the-art in fear recognition, achieving a recognition rate of up to 96.33% for the subject-dependent model.

## 1. Introduction

Physiological monitoring technology has received a great deal of attention during recent years from both academics and industry because of its potential applications in relevant areas such as healthcare and human–machine interaction [1]. Additionally, emotion recognition is a multi-disciplinary field of knowledge with links to many research areas, such as psychology, neuroscience, signal processing and machine learning [2].

Most emotion recognition systems in the literature focus on classifying emotions from a general-purpose point of view by detecting a set of emotions without considering the gender perspective [3]. However, targeting the identification of a single emotion that could be related to a specific situation and considering gender-related particularities could result in a more accurate system. This last assertion is based on the idea that women more accurately recognize nonverbal communication or emotional prosody [4] and are more sensitive to emotional expressions in interpersonal interactions [5]. These concepts are not considered in any current emotion recognition system using physiological signals presented in the literature. Currently, to the authors’ knowledge, there is no automatic detection system that has been developed to identify different critical social situations, such as gender-based violence. Within this context, a gender-specialized fear detection system could be used to trigger a protection protocol that could include a connection to a trusted circle or even to law enforcement agencies, in order to provide the necessary help. This kind of system would be in line with the fifth Sustainable Development Goal provided by the United Nations, which highlights the need for gender equality as a required foundation for a peaceful, prosperous and sustainable world.

An important consideration when designing emotion recognition systems using physiological signals and aiming, as a long-term goal, to be able to apply the systems to real-life scenarios is an inconspicuous appearance. For instance, there are many sensors, such as electroencephalographs or electrooculographs, which are not ready to be integrated within a current commercial wearable device; e.g., a wristband or a pendant. An emotion recognition system that is intended to detect emotionally critical situations on a daily basis should be designed not to draw people’s attention. Therefore, such systems should be designed considering a wearable form, which implies the integration of the most inconspicuous sensors [6].

On this basis, in this paper, we aim to design a fear recognition system using a reduced set of physiological signals from sensors that can be located in many of today’s smartwatches or activity bracelets. Reviewing the sensors that are available on the market that fit the wearable integration requirement and have relevance in the emotion recognition field, the authors selected the electrocardiogram (ECG), skin temperature (SKT) and galvanic skin response (GSR) sensors [6]. The main focus of this paper is emotion recognition for women, and we propose systems that aim to protect this specific population. According to [7], a significant difference in emotional response exists between women and men. Consequently, only data from female participants were considered to train the fear recognition system. Moreover, a discrete emotional binary mapping into a specific dimensional emotion model is used to provide a mechanism to reduce emotional self-assessment bias. The main contributions of this paper are as follows:

- A specialized emotion recognition system based on binary fear classification and only three physiological signals (ECG, SKT and GSR) is presented.
- A fear emotion detection system is pursued, focusing only on women volunteers. This approach is due to the different emotional responses found between women and men in the literature.
- A specific mapping between the discrete emotion of fear and a well-known dimensional emotion model is applied and used for our specialized emotion recognition system.
- The emotion recognition system considers linear (temporal and frequency), and non-linear feature extraction for the physiological signals. To the authors’ knowledge, this is the first time that the combination of these features with a reduced set of physiological signals and a fear binary classification considering discrete and dimensional information about emotions has been proposed.
- The proposed system is validated through an open emotion recognition dataset (MANHOB [8]) adapted to our use case. Experimental results show a fear recognition rate of up to 96.33% and 85.00% for subject-dependent and subject-independent models, respectively. The performance of the subject-dependent model outperforms any other subject-dependent approach in the literature. To the authors’ knowledge, this is the first time that a subject-independent model considering specific fear binary mapping, different emotional models (dimensional and discrete) and three physiological signals has been presented. The results are limited by the number of participants in the original MANHOB dataset (30 people). This initial limitation resulted in only 12 participants being considered for our experiments due to the selection of only women from MANHOB, as stated above. At this point, this limitation cannot be addressed because there is no other public dataset available in the literature that fits our use case.

The rest of the document is structured as follows. Section 2 discusses related work in emotion recognition and provides background about emotion theories and databases considering physiological signals within the field. The proposed model is detailed in Section 3. Section 4 summarizes the different tools considered and particularities related to the specific validation presented. Experimental results are discussed in Section 5. Finally, conclusions and future research directions appear in Section 6.

## 2. Background and Related Work

Research into emotion recognition using physiological signals has increased in recent decades. Physiological signals are a valuable source of information related to the human emotional context; this is because the autonomous nervous system controls these signals, meaning that they cannot be manipulated in any way by human will [9,10].

As stated above, the proposed system should be able to classify a single emotion (fear) felt by the user from the analysis of physiological data. To this end, the intelligence engine should be first designed and trained according to a database of physiological signals captured during the reception of pre-tagged stimuli.

A deep understanding of human reactions to stimuli and emotion inference is required to achieve this goal, as well as the knowledge of the different available emotion theories and models. The selection of an adequate emotional model is especially relevant because it is related to the labeling of the emotion felt by the individual. Note that the better the emotion labeling during the database generation, the better the performance of the intelligence engine. The rest of this section discusses emotion theories and models, as well as databases for emotion recognition using physiological signals.

### 2.1. Emotion Theories and Models

Two main emotion theories have been presented in the literature. These are categorical (also called discrete) and dimensional models. Ekman [11,12] and Russel [13], respectively, are mainly responsible for the adoption of these models.

Categorical models are based on the concept of basic emotions, which are cross-cultural and universally recognized. These emotions correspond with the primary response to stimuli that triggers an immediate, innate and universal reaction that is highly related to survival [14]. Note that, of these basic emotions, fear is included in all the proposed sets. Categorical models include some important shortcomings: (1) the same basic emotions are not present in every culture and (2) the analysis of complex emotions is a challenging task. Although some authors proposed families of emotions, which cover distinct differences depending on the person’s temperament [15], another fine-grained classification model is required to match human emotions with sensed data during the reception of stimuli unequivocally.

In this sense, quantitative scales for different aspects of emotion allow the creation of a dimensional emotion space model. Wundt [16] was the first to introduce two dimensions to differentiate emotions: pleasant–unpleasant (valence) and low–high intensity (arousal). Unlike qualitative emotional information provided by discrete models, dimensional models give specific quantitative metrics regarding affective states [17,18]. Thus, dimensional models have been shown to be useful when designing emotion recognition systems powered by machine learning. This is because the labels used for training are more specific, resulting in better intelligence systems.

Regarding the identification of specific emotions using both theories, Albraikan et al. in [19] tried to diminish the possible bias effect by combining both models and mapping arousal and valence dimensional space into a specific discrete emotion. However, their model was not able to capture the difference between fear and anger. In this regard, Demaree et al. in [20] affirmed that a three-factor model (arousal, valence and dominance) is required to identify an affective state. They compared the fear–anger distinction using the circumplex model [21] and the pleasure, arousal and dominance (PAD) model [22]. As a result, Demaree et al. stated that only dominance can disentangle such emotions as fear and anger, with low and high dominance, respectively. For these reasons, instead of considering discrete emotions directly or only using a two-dimensional model (arousal and valence), the fear detection system proposed in this paper maps the PAD dimensional space into a discrete binary emotion problem. Thus, fear-related emotions are defined by low valence, high arousal and low dominance.

### 2.2. Databases for Emotion Recognition Using Physiological Signals

A database for training an emotion recognition system is composed of three main elements, apart from the indispensable volunteers:

- Stimuli to evoke different emotions in volunteers;
- Emotion labels for every stimulus received;
- Physiological variables measured during the reception of stimuli.

From these elements, the latter two (labels and physiological data) are the inputs required for the machine learning training task. Labels can be implemented by experts or self-reported by the volunteers. Note that, due to different temperaments or traits, a non-negligible dispersion in the labeling is introduced. However, this dispersion can also be due to the inherent difficulty of identifying the emotion felt and its translation to the dimensions asked (e.g., arousal, valence and dominance). This labeling problem is challenging for the development of subject-independent emotion recognition models. That means that the protocol for database generation should address in detail the labeling process. In this regard, there are accepted labeling methods that have been proposed by experts in emotion elicitation to help in this task. For example, self-assessment manikins (SAMs) [23] are commonly referenced, although they present male tendencies, which can affect the interpretation of emotions for women. That means that SAMs should be improved to include a gender perspective. Moreover, stimulus interpretation is strongly volunteer-dependent [15] and affected by gender. Thus, customization has been implemented, as done in [6], in which Schmidt et al. concluded that human emotions are subject-dependent. After performing a detailed review of wearable-based emotion recognition, they affirmed that an emotion recognition subject-independent model could be deployed but that, at some point, user customization will be necessary to improve the system.

Within the emotion recognition community, different publicly available datasets consider physiological signals. The most common are MIT [24], DEAP [25], MAHNOB [8], DECAF [26], ASCERTAIN [27], and WESAD [28]. In [29,30], the authors performed a detailed analysis of some of these databases and provided conclusions about their methodologies and emotional recognition approaches. These open databases provide a solid benchmark for current and future emotion recognition systems based on physiological signals. The proposed emotion recognition solution in this paper was validated using MAHNOB [8], as this database includes more self-reported information regarding emotion labels than others. However, the system could be validated using any other database with the required self-report information; i.e., emotional discrete and PAD metric reports, as well as gender information and data from the three physiological sensors considered. It should be noted that there are currently no balanced open databases for the emotion of fear using physiological signals and considering the gender perspective.

### 2.3. Related Work in Emotion Recognition Systems Using Physiological Signals

There are three main aspects of emotion recognition systems using physiological signals affecting system performance: data segmentation, feature extraction and data classification.

#### 2.3.1. Data Segmentation

Focusing on data segmentation, most of the emotion recognition systems in the literature use data processing windows to treat and analyze the acquired data. Thus, data segmentation is characterized by window-related aspects, such as their temporal and frequency resolution and emotional latency. On the one hand, the temporal resolution has a direct relationship with frequency resolution. This is due to a specific frequency resolution needing to be guaranteed to extract useful emotional information for some physiological features [31]. On the other hand, emotional latency is related to the fact that a person does not experience the same physiological response (emotion) during the entire reception of a stimulus [32,33]. This last aspect definitively affects the system performance because it is related to the possible incorrect labeling of the input samples.

Reviewing the literature, different studies have applied data segmentation over the physiological recollected data. For instance, Zao et al. [34] presented a similar use case to that in this work but using their stimuli instead of an openly available database and classifying arousal and valence rather than a unique emotion. They omitted the beginning and the end of the experimental measured data fir each stimulus, using the smallest known temporal window (16 s). As a result, they obtained up to 75.56% accuracy on average for a subject-dependent approach in a four-quadrant classification problem (arousal–valence). Kanjo et al. [35] performed a subject-dependent system using a constrained field experiment, a wearable system, and also omitted the first and the last seconds of each trial. They tested different window sizes and obtained up to 87.30% accuracy on average by using deep learning techniques in a five valence level classification problem. In [36], Hassan et al. proposed a conjunction of conventional machine learning algorithms and a deep belief network. They performed an analytical study over the DEAP [25] database and concluded that induced emotions are stronger in the final part of the stimuli. Thus, they trained the system using only the segmented 20 last seconds of the measured data, obtaining up to 89.53% accuracy for a subject-independent model in a five discrete emotion classification problem (happy, relaxed, disgusted, sad and neutral). One of the latest emotion recognition systems, WESAD [28], uses 60 s segmented windows. The researchers obtained up to 93.12% accuracy for a subject-independent model by using linear discriminant analysis in a binary stress detection problem. Note that stress is not considered to be an emotion. Analyzing all these related works, it should be noted that the effects of data segmentation were not discussed. For instance, a rationale was neither given regarding time and frequency resolution requirements in physiological-based emotion recognition systems nor for physiological response delay times. The latter is key when dealing with slow-changing physiological signals, such as GSR, which is known to have time-varying responses from 1 to 30 s based on the type of stimulus [37].

#### 2.3.2. Feature Extraction

Regarding feature extraction, the best detection capacity results for emotion recognition systems using physiological signals were obtained by Rubin et al. in [38]. They applied data segmentation together with linear and non-linear feature extraction techniques, rather than simply using temporal and frequency-based linear features. They used a wearable electrocardiogram and collected data from individuals suffering from panic disorder in an ambulatory setup. As a result, they obtained up to 97.20% accuracy on average by using linear and non-linear feature extraction, in combination with random forest classification, in a panic-based binary classification subject-independent system. Note that, within this system, emotional labels such as PAD were not included, as the researchers were not inferring an emotion but distinguishing between pathological and non-pathological states associated with panic disorder. Another recent work [19] used multidimensional dynamic time warping as a non-linear technique to deal with physiological dynamics. They obtained up to 94.00% and 93.60% accuracy for a valence and arousal subject-independent model by using all signals from the MAHNOB database. Most emotion recognition systems based on physiological signals and using accepted emotional labels are based on conventional temporal and frequency linear feature extraction. Thus, the combination of the three domains (temporal, frequency and non-linear) should be extended in the literature. This approach might be exploited to gain a better understanding of the physiological variations and changes concerning the self-reported metrics used as labels within these types of systems.

Another step commonly implemented after feature extraction is the application of feature selection techniques to reduce the redundant information and the dimensionality of the problem. In this regard, previous results obtained in [30], in which the model performance was compromised, led the authors of this work not to include this step in the current study.

#### 2.3.3. Data Classification

Related to data classification, different machine learning and deep learning techniques have been used for emotion recognition systems in the literature. In this context, the most used machine learning algorithms were the support vector machine (SVM), k-nearest neighbor (KNN), ensemble classifiers (ENS) and random forests [1,6]. These classifiers provide low computational complexity and straightforward implementation on low-resource devices. Note that, regardless of the classification approach, the preprocessing and feature extraction might be the most computationally intensive part of the inference stage. Moreover, in the last years, the development of open-source deep learning frameworks for constrained devices, such as TensorFlow Lite, are leading to the use of neural-based inference approaches [39].

To the authors’ knowledge, there is only one emotion recognition system in the literature that proposes a specialized fear binary recognition approach [40] and obtains a fear accuracy below 90% by using the same discrete and dimensional emotion models combination as ours. They validated their system based on DEAP but using all signals, including an electroencephalogram. Moreover, they did not consider any constraint related to data segmentation, frequency resolution, feature extraction, storage or complexity. The other works mentioned above [28,34,35,36,38], which were based on different emotional detection use cases, all considered wearable sensors; i.e., systems that can be integrated following an inconspicuous factor form. However, none of them included self-reported dominance by the volunteers in the labeling problem. This last fact has a key impact on the distinction of fear and therefore on the system, as stated in the previous section. Besides, the authors in [35,36] considered deep neural networks and other techniques that are not feasible for low-power embedded integration or could imply high computational complexity. Another key aspect which was not studied within these references is the effect that a balanced data distribution has on both subject-dependent and subject-independent models, as well as the effect on gender distinction. These facts are directly related to the type of stimuli used during the emotion elicitation and they have a key impact on the generation of any subject-independent model.

This work proposes a specialized fear recognition system for women based on only three physiological sensors and the use of a discrete emotion binary mapping process into three emotional dimensions (arousal, valence and dominance). Analyses regarding the balanced data distribution and its repercussions for the classification metrics are also provided. Additionally, two different systems, subject-dependent and independent, are presented and analyzed.

## 3. Proposed System

The proposed emotion recognition solution consists of two main stages: data segmentation and data processing. These stages follow the typical processing chain employed in emotion recognition [1,6]: segmentation, preprocessing, feature extraction and data classification. The presented approach benefits from the state-of-the-art methodology to provide a system that meets the following requirements:

- The system must be focused on fear emotion recognition.Thus, the physiological dataset used must address the elicitation of fear. This concern affects the dataset selection and processing.
- The emotional labeling of the stimuli must encompass dimensional and discrete self-reported emotional information. This fact is related to the classification strategy and methodology used.
- The type and the number of sensors impose a limitation related to the diversity of information available. These sensors must be chosen to be applicable for current wearable device integration.

**Figure 1:**
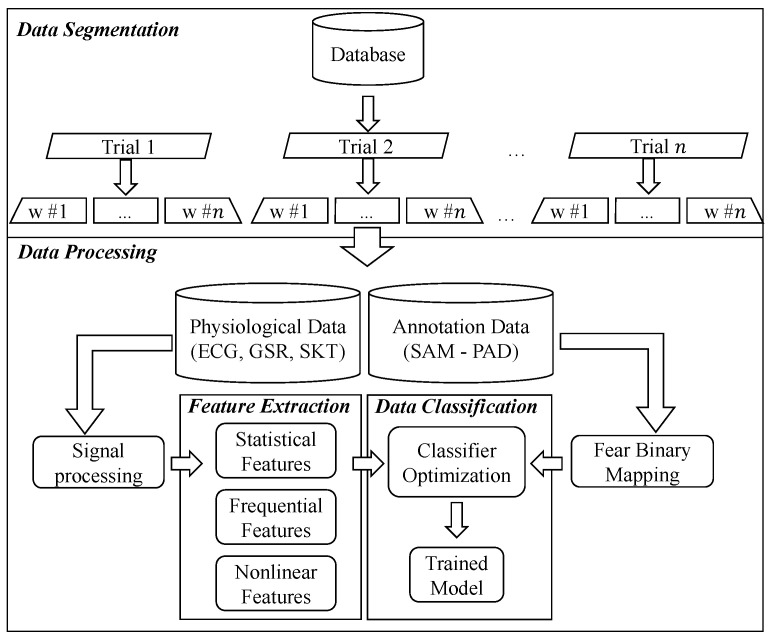
Overview of the proposed fear recognition system employing physiological sensor data and the pleasure, arousal and dominance (PAD) dimensional approach emotion labeling. The latter is fed into the fear binary mapping procedure. ECG: electrocardiogram; GSR: galvanic skin response; SKT: skin temperature; SAM: self-assessment manikin.

Figure 1 shows an overall description of the proposed fear recognition system. It includes the usual steps in the processing chain discussed above, from the analysis of an available dataset of physiological signals to raw data preprocessing, feature extraction and emotion classification. Note that w#n denotes the different windows obtained after data segmentation application. The next sub-sections describe each of the elements in this processing chain.

### 3.1. Data Segmentation

The system starts by considering a database in which data captured from three physiological sensors (ECG, GSR, and SKT) are included, while stimuli are shown to volunteers and the emotions felt are labeled according to the PAD space. This database contains information from several volunteers. If only data from one volunteer are considered to generate the system, a subject-dependent approach will be produced. Conversely, if data from all the volunteers are considered, a subject-independent approach will be generated. Both approaches are analyzed in the next sections.

As stated in Section 2.3, data segmentation or window-based methods are used to extract emotion-related information concerning time instants. Following the accepted data segmentation procedures in the literature, a window-based methodology is adopted in this work. Based on the sampling frequency of the different sensors, an appropriate window length is chosen to ensure that the frequency resolution is sufficient to deal appropriately with all the frequency-based features. Data are encapsulated in fixed time slots to be processed during the next data processing stage. The size and number of windows are configurable parameters in the proposed system, together with the window overlapping, allowing researchers to search for the best physiological option considering both the time and computational complexity.

### 3.2. Data Processing

The segmented data (windows) obtained during the previous stage are preprocessed to eliminate noise and other non-useful components for the next steps. Thus, the overall signal quality is improved by denoising filters, focusing on their specific physiological characteristics. Specifically, the raw ECG signal is subjected to a band-pass FIR filter through a low and high pass filtering cascade to ease complexity [41]. Afterwards, automatic gain control is applied to limit the signal and enhance the peak detection. For the GSR and SKT signals, low-pass FIR filters are employed to remove high-frequency noises. Next, features of interest are extracted from the noise-free physiological signals, meaning that a lightweight binary classification algorithm can be trained to predict the fear occurrence in future samples.

The data processing strategy presented in this section was designed to generate (train) the machine learning model. However, the architecture can be adapted for the prediction of samples during the production stage. To this end, the task of training the model should be replaced by applying the inference stage of the previously trained model, resulting in the fear prediction of the segmented input. The rest of this section focuses on discussing the feature extraction and data classification techniques used.

#### 3.2.1. Feature Extraction

This stage plays a key role in any classification system, but this is even more applicable when considering such complex inputs as physiological signals. Thus, our proposal considers features from the three main groups: time-domain, frequency-domain and non-linear features.

Time-domain features have the lowest computational complexity, providing useful information about stationary signals with linear dynamics. Frequency-based features are also widely applied to obtain power spectral densities in specific frequency bands for physiological signals, such as ECG and GSR [42]. These frequency-based features usually require a higher computational effort than time-domain features. Non-linear features are useful to find trends in non-linear and non-stationary signals, with promising solutions presented in the literature, but imply complex implementations. Our proposal considers the set of 48 features presented in Table 1. Specifically, 25 features for ECG (two in the time domain, nine in the frequency domain and 14 non-linear features), 17 features for GSR (six in the time-domain, three in the frequency-domain, and eight non-linear features) and six features for SKT (four in the time-domain and two in the frequency domain) are included.

All the time-domain and frequency-domain features considered in our model are based on accepted, well-known physiological literature dealing with emotional-related features [31,43,44]. The raw ECG signal is subjected to R-peak identification to determine the inter-beat intervals (IBI) and extract a valid heart rate estimation and heart rate variability-related parameters. The GSR signal consists of two main components: tonic (also called skin conductance level) and phasic (also called skin conductance response). The latter reflects both event-related skin conductance responses (ERSCRs) and non-specific responses (NSSCRs). As stated in [37], characteristics regarding rising time and peak amplitude differ for both ERSCRs and NSSCRs. Our proposal includes a moving median filter to separate the tonic and phasic components and a trough-to-peak standard peak detection method, which is characterized by low computational complexity. For the SKT signal, no particular digital signal processing, other than the previous filtering, is applied before feature extraction.

The non-linear features considered in our model are based on recent publications that included these metrics in an emotion recognition system [38,45]. The calculation of most of the non-linear features considered requires the recurrence plot (RP) [46] to be obtained based on the phase space trajectory followed by a specific raw physiological signal. RPs are a bidimensional plot showing, for each moment in time $t_{1}$, the number of times that the phase space trajectory of the signal visits the same area in the phase space at time $t_{2}$, with both $t_{1}$ and $t_{2}$ represented on the horizontal and vertical axis, respectively [47]. The RP calculation requires some parameterization; i.e., the time delay *T*, the embedding dimension *M* and the threshold distance *E*. The authors estimate *T* and *M* using mutual information [48] and false nearest neighbor [49], respectively, and define *E* as 10% of the average phase space diameter of observations [50]. A recurrence quantification analysis [51] is applied to quantify valuable information by exploring the obtained RPs, which provides the different extracted non-linear features.

#### 3.2.2. Data Classification

As introduced in Section 2.3, the proposal only considers lightweight binary supervised classification algorithms. In this line, a design space exploration was developed, and this is described in Section 5. This was accomplished by applying a set of low-complexity algorithms in the emotion recognition field; i.e., SVM, KNN and ENS. Other classification algorithms could be considered for the proposal without loss of generality, also including key factors such as the time complexity and the necessary resources.

Most classification algorithms include hyper-parameters that affect the algorithm performance [52], which should be fitted before performing the training stage. In this regard, the proposal considers Bayesian optimization to minimize the misclassification rate over iterations, supported by a cross-validation strategy. Specifically, a sequential model-based optimization technique is included.

Data labeling is a critical task when designing a supervised classification strategy; i.e., assigning the value for the property to be predicted is for a given input sample. As expected, this task definitively affects the classification algorithm because it determines the correctness of the information from which the algorithm will learn. On this basis, a binary label was adopted for the fear emotion; i.e., 1 (positive class) if the sample referred to fear and 0 otherwise. As input labels in the database were given in the three-dimensional PAD space, a binarization stage was required to map the discrete fear emotion into the specific PAD quadrant. That meant that samples with low valence, high arousal and low dominance were labeled as the positive class, whereas the rest of the combinations referred to the negative class (see Section 2.2). Note that the distinction between low and high values for the three dimensions was made by equally dividing the SAM scales from 1 to 9 [23]. This proposal could be extended to detect other emotions by binarizing the labels in the database accordingly.

## 4. Tools and Methods

The proposed emotion recognition system, which was fully encoded by the authors under MATLAB®2019b, was experimentally evaluated using the MAHNOB dataset [8]. This lab-based emotion recognition dataset includes data from several volunteers who observed 20 one-minute video clips. The recorded physiological responses were acquired using the Biosemi active II system. The acquired data included ECG, GSR, respiration amplitude, SKT, electroencephalogram, eye gaze and face and body videos. A total of 30 adults participated in the original experiment, of which 17 were women and 13 were men. However, only 12 of the female volunteers were recorded without failures and permitted the publishing of their data. As stated in Section 1 and Section 2, as we aimed to develop a specialized fear recognition system for women, only the women participants in MAHNOB were considered for the experimentation. That means that the results presented in this work could be limited by the reduced number of participants during the experimentation for the models proposed. However, to the authors’ knowledge, there is no other available dataset that fits our case in the literature.

The MAHNOB database generation considered the emotional recoveries of volunteers between stimuli. That means that, before watching any emotional video, different neutral clips were shown to the participants. This process was used to recover a basal physiological level, decrease the emotional bias after experiencing an emotion and handle physiological intra-subject differences. All recorded responses for each stimulus contained 30 s of data at the beginning and the end of the slot corresponding to this recovery process. These 60 s periods in the experimentation were eliminated.

In response to the data segmentation described in Section 3, and with the aim of obtaining a low processing time in each window, a minimum fixed temporal window size was selected. This decision was led by frequency resolution restrictions imposed by the features to be extracted. Thus, a fixed window size of 20 s with a 10 s overlap was applied over the 20 trials or audiovisual stimuli. This decision directly affected two key factors. On the one hand, the frequency resolution was set to 0.05 Hz/bin, which was enough to ensure the frequency precision needed. Note that the sampling frequency for all the sensors in this database was 256 Hz. Thus, this window size provided the best trade-off between the temporal complexity and necessary resources within the window. Note that the bigger the window, the higher the frequency resolution, but the larger the memory required to store the data and the time to process it. On the other hand, the limitation of dealing with this window length and an ERSCR duration greater than 20 s was taken into account. Note that, as stated in Section 2.3.1, ERSCRs may vary between 1 to 30 s, although the use of a 50% overlap allowed for a balanced trade-off between the amount of ERSR information lost and memory requirements. Therefore, each trial was segmented into a total of five windows that had the same class or label. Note that the greater the overlap, the more operations needed to be done within a specific time interval. Concerning the storage of the acquired signals into an embedded platform—for instance, assuming a maximum width of 32-bits for each data point—the parameters set would lead to a 60 KB memory requirement (256 samples per second × 20 s × 3 sensors for 32-bit samples). This storage space could be provided by the current system-on-chips that are used for many wearable devices. Nevertheless, these requirements are application-driven and can be modified based on the embedded platform capabilities.

As stated in Section 2.1, the authors considered the MAHNOB subjective self-reports from volunteers according to their experience during the visualization of stimuli. According to these labels, a fear binary mapping was done as specified in Section 3.2.2. The machine learning metrics used to assess our model performance were accuracy (ACC), the area under the curve (AUC) [53], geometric mean (Gmean) and F1 score. The former is calculated as

(1) $$ACC=\frac{TP+TN}{TP+TN+FP+FN}\;,$$

where TP and TN are the true positives and negatives, respectively, and FP and FN are false positives and negatives, respectively. AUC is given by

(2) $$AUC=P(X_{1}>X_{0})\;,$$

where $X_{1}$ is the score for a positive instance and $X_{0}$ is the score for a negative one. Gmean is calculated as

(3) $$Gmean=\sqrt{sensitivity*specificity},$$

where sensitivity is the true positive rate and specificity is the false negative rate. The latter is determined by

(4) $$F1=\frac{2*TP}{2*TP+FP+FN}\;,$$

where TP, FP and FN are the true positives, false positives and false negatives, respectively. Regarding the validation procedure, the subject-dependent and subject-independent models were validated based on a stratified k-fold cross-validation schema (k=5). Note that, for the subject-dependent models, the mean of all metrics for all volunteers and the mean absolute deviation (MAD) were calculated. Specifically, the subject-independent models were divided into training–validation–testing sets, employing a leave-one-subject-out (LOSO) strategy. The latter allowed us to study the performance of various subject-independent systems trained with different subject combinations and tested with a single volunteer about whom the system had no information.

## 5. Results and Discussion

This section presents the experimental analysis of both subject-dependent and subject-independent models for a specialized binary-fear recognition system based on a reduced set of physiological signals for women.

### 5.1. Subject-Dependent Analysis

**Figure 2:**
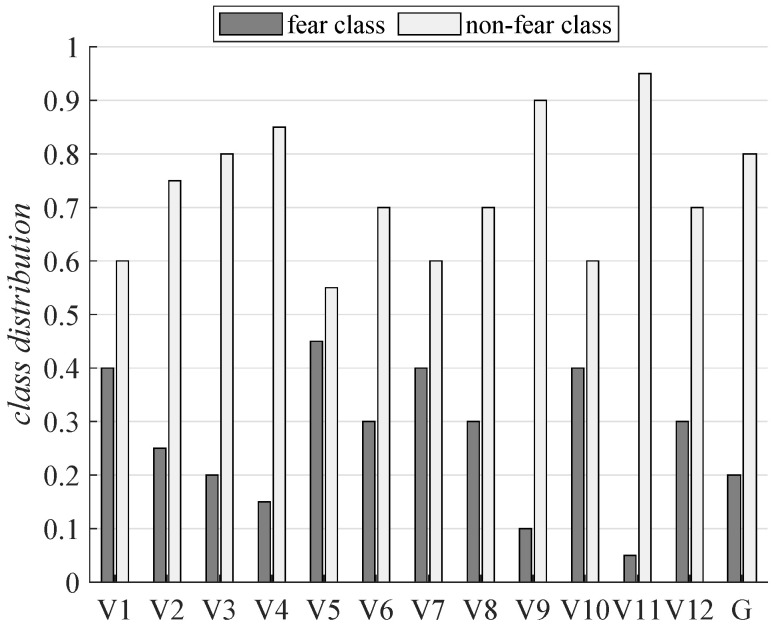
Subject-dependent class distribution for binary fear mapping over the subjective self-reports in MANHOB, and the original intended class distribution of the experiment.

Before considering the classification problem, an exploratory data analysis over the different extracted physiological features was performed to check for outliers and verify that all generated data were physiologically in-range. These models were adequately scaled but not subjected to any normalization. Note that, as stated in the previous section, physiological recovery time-slots were omitted. After performing the fear label binarization in MAHNOB, the obtained distribution was analyzed for all female volunteers, resulting in asymmetry. That meant that the appearance of fear labels was not uniform for all of the participants. Thus, Figure 2 shows that 60% of the volunteers had more than 30% of binary-fear labels, whereas the rest of the volunteers were below that amount. Note that, in this figure, the notation Vx means volunteer *x*, with x∈1…12, and the notation *G* refers to the original binary-fear distribution of the experiment (the actual number of stimuli intended to elicit fear; i.e., only 20% of the total amount of videos). This unbalanced situation is especially relevant for V11, with only 5% of fear data. That means that the interpretation of stimuli was strongly volunteer-dependent, as expected. This makes the selection of general stimuli which elicit fear difficult. The asymmetry observed also affected the performance metrics to be considered. Thus, ACC was not enough to assess model performance [54]. Therefore, more robust metrics were needed, such as the AUC, Gmean and F1 score. Note that the class distribution does not imply that two volunteers with the same distribution reported the same stimulus as inducing fear. Thus, the performance of each of the subject-dependent models generated was subjected to the disentanglement of the physiological fear-related patterns of the subject through the binary fear mapping obtained through self-reports.

Table 2 shows the validation performance metrics and dispersion for the different light-weight classification algorithms selected for the generation of each subject-dependent model for all volunteers. After analyzing the results, it can be observed that there was no strict dependence relationship between the class distribution and performance. Nonetheless, the performance of the models was directly affected by the type of classifier used. Moreover, as stated above, another key factor that could have influenced performance was related to the alignment of subject-dependent physiological patterns and the binary fear mapped labels obtained. Furthermore, the usage of Gmean and F1 score allowed us to distinguish the low-performance models from the higher-performing models more robustly.

**Figure 3:**
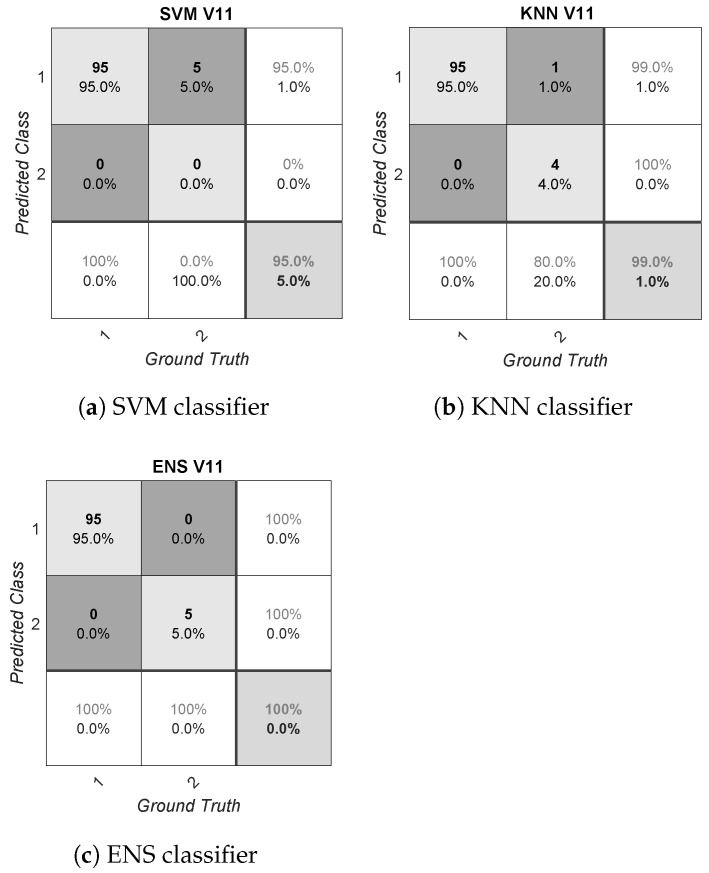
Confusion matrices for a subject-dependent model in V11, detected as a problem in asymmetry.

**Figure 4:**
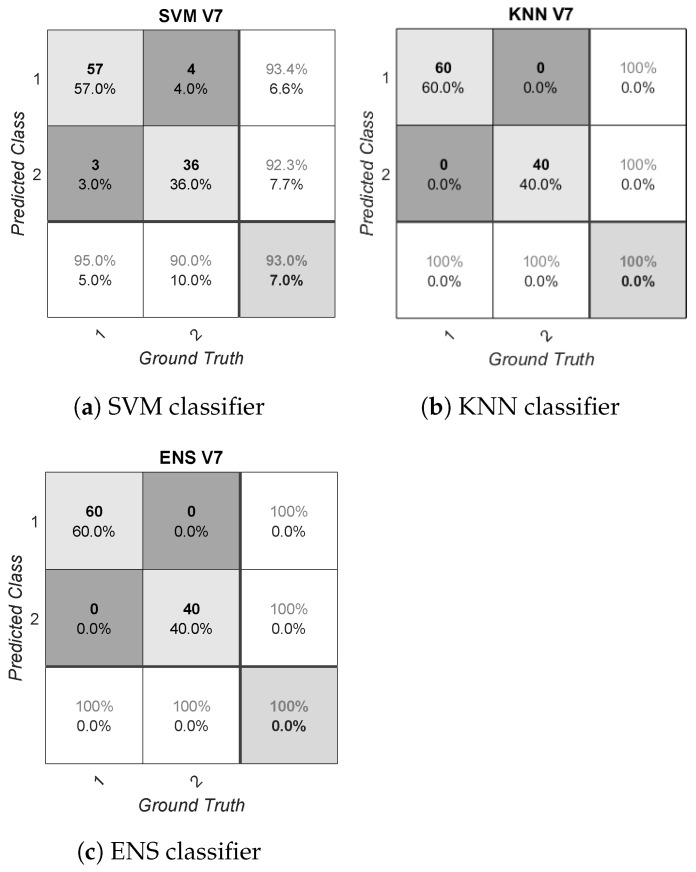
Confusion matrices for a subject-dependent model in V7.

The presented results could be biased due to the reduced amount of data available (100 samples per volunteer, with 20 videos and five windows each), as well as due to the asymmetry detected (imbalanced data). Focusing on asymmetry, this problem is especially relevant in V11, as discussed before. The effect on performance due to asymmetry for this volunteer is shown in Figure 3. This figure provides the confusion matrices for V11 after applying all the algorithms. Conversely, the confusion matrices of the volunteer V7 are also shown in Figure 4. This volunteer was considered to show the best performance overall; i.e., considering the different metrics for the three classifiers applied. In these figures, the positive class is represented by the number two, and the negative class is represented by the number one. The rows correspond to the predicted class and the columns correspond to the true class or ground truth. From left to right and from top to bottom, each confusion matrix shows the TN, FP and false omission rates. The next row shows the FN, TP and precision rate. The last row shows the false-negative rate, specificity and overall accuracy. Note that the rest of the confusion matrices for each subject-dependent model generated are shown in Appendix A.

After analyzing these confusion matrices, the performance of the algorithms in V11 was also found to be asymmetric. Thus, for instance, SVM provided a high accuracy, at up to 95.00%, but this metric was biased by the reduced number of samples of this volunteer within the positive class (only five samples). In this case, the calculated Gmean and F1 metrics results were 0.00% due to the zero positive predicted rate, and the AUC was 50.00%, showing that this classification model performed no better than random guessing. The behavior shown by SVM matched the usual unreliable performance of this algorithm for extremely imbalanced distributions; that is, SVM is oriented towards the majority class to optimize the error rate during the training stage. On the contrary, boosting algorithms usually provide a better behavior for imbalanced distributions, as shown by ENS for this case. Nevertheless, this imbalanced situation should be avoided during the database generation, and the quality and diversity of the stimuli considered should be improved. In the case that this situation is not addressed during the database generation, the bias generated in performance could be partially solved by selecting an adequate classification technique, as discussed above. However, the lack of information from one of the two classes cannot be solved, resulting in a possible incorrect classification for future samples [55]. Another possible approach to deal with this problem is based on the application of data augmentation techniques or weighted classes. Some of these techniques have been already used in [30] by the authors. Conversely, in the case of V7, the system showed 40.00% positive class information, which translates into a better SVM performance. KNN and ENS continued to outperform SVM due to the reasons stated above for the error rate optimization of this classifier.

To the authors’ knowledge, the results obtained for the subject-dependent fear detection model proposed in this paper outperform the state-of-the-art approaches. Thus, the only binary fear recognition system known in the literature [40] with an accuracy above 90% was surpassed, as our approach reached 96.33% accuracy. Other related works within the wearable emotion recognition field were also outperformed, such as the proposals in [34,35], providing up to 75.56% and 87.30% accuracy values. The valuable work in [38] provided a similar accuracy to our approach, with up to 97.20% accuracy. Note that the works in [34,35,38] were for a different use case, and the performance metrics shown should be considered as an indication of usual performance metrics within the field. Note also that this comparison could be biased due to the usage of ACC in these papers instead of more robust metrics as Gmean or F1.

### 5.2. Subject-Independent Analysis

Another possibility would be to generate a subject-independent approach to obtain a global model for the entire population. This option avoids customization for each user, facilitating the delivery task and making the system more user-friendly. In this case, the combination of all volunteers would substantially increase the number of samples. Thus, there would be more data to train and test the models, which is especially valuable in the emotion recognition field. However, the subject-independent approach aimed to find a universal model for the global population, presenting a challenging task for our system considering the observed dispersion in emotion labeling for different individuals. The possibility of generating a subject-independent system is still an open question that requires a larger data set combined with an improved labeling methodology and a balanced selection of emotion-eliciting stimuli.

Focusing on our use case, the combination of all individual samples resulted in a bigger dataset with 1200 samples (20 videos × 5 windows each × 12 volunteers). The physiological signal ranges differed for different individuals due to the nature of each individual and the differences in the measurement set-up (e.g., ambient temperature). Therefore, the data from each volunteer should be normalized. To this end, the authors considered the z-score method. Once the database was normalized, the binary-fear recognition system was generated using a k-fold cross-validation schema and a LOSO testing methodology, as stated before in Section 4.

Table 3 shows the performance metrics for each classification algorithm in the generation of the subject-independent model. Note that the training of these models was performed using all volunteers except the one used for testing in each iteration (unseen test data); i.e., a total of 12 subject-independent models were generated and tested.

**Figure 5:**
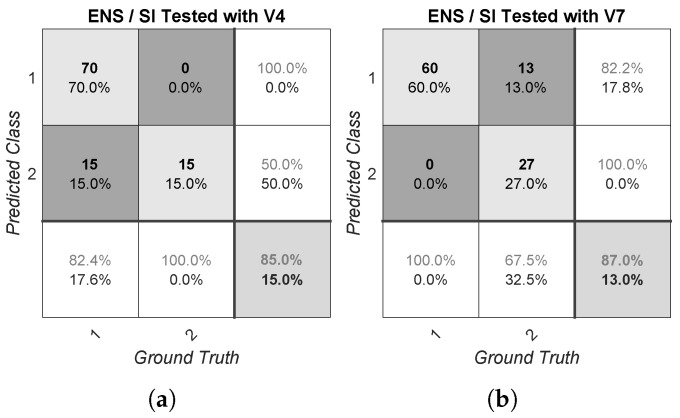
Confusion matrices for ENS classifiers and tested volunteers (unseen data) over their respective subject-independent models: (**a**) tested V4, (**b**) tested V7.

After analyzing this table, the best results were also provided by ENS, with the highest averaged performance metrics (81.20%, 72.84%, 56.11%) for the AUC, Gmean and F1 score. On the contrary, SVM also provided the worst performance in general. The differences between all the subject-independent models generated should be noted. For instance, the best model achieved a Gmean of up to 90.75% when testing with V4 and training with the rest of the volunteers, and the worst model provided a Gmean of up to 45.82% when testing with V3 and training with the rest of the volunteers. This fact emphasizes the need for a larger and more balanced data set to deal with these problems. Regarding the F1 score, a high variability can be observed between the different models. By definition, this score is a weighted harmonic mean between precision and recall, which leaves TNs out of the equation. This fact is key when presented with a very low positive incidence, but a high F1 score does not necessarily imply a better performance of the system. For instance, the confusion matrices of two subject-independent tested models for the ENS classifiers are shown in Figure 5 for V4 and V7 with F1 scores of up to 66.67% and 80.60% respectively. Based on the pursued fear recognition application, it could be more convenient to have a misclassification for the FPs than over the FNs. Therefore, comparing the F1 score for different subject-independent models should be accompanied by the requirements and needs of the application. Note that both of the explained examples did not show a perfect classification performance. The rest of the confusion matrices for each subject-independent model generated are shown in Appendix A.

The best subject-independent model generated corresponded with the only specialized fear recognition system in the literature, which considered a subject-dependent model (below 90% accuracy). This fact is especially relevant due to the advantages of the independent approach and the usage of only three physiological signals. Other related works correspond with the result obtained for a subject-independent approach as well. For instance, the works in [28,36] provided up to 93.12% and 89.53% accuracy values, while that in [19] showed up to 94% accuracy. Note that these other related works are based on a different use case—i.e., not binary-fear recognition—and the performance of their models was defined by the accuracy metric, which is not enough to assess model performance, as sated previously.

## 6. Conclusions and Future Work

A specialized fear recognition system for women based on a reduced set of physiological signals has been proposed in this work. This system is characterized by the usage of three physiological signals (ECG, GSR and SKT), a binary fear mapping strategy based on self-report information, low computational complexity binary classification algorithms and the conjunction of linear and non-linear features.

The authors evaluated the system using the MANHOB database with two approaches; i.e., subject-dependent and subject-independent models. Focusing on the subject-dependent approach, the proposal achieved a fear accuracy recognition rate of up to 96.33% and a Gmean of up to 95.51% for 12 volunteers. Focusing on the challenging subject-independent approach, the proposal achieved an average fear accuracy recognition rate of up to 76.67% and an average Gmean of up to 72.84% considering a LOSO testing methodology. Note that the best subject-independent model provided a fear accuracy recognition rate and a Gmean of up to 85.00% and 90.75%, respectively.

Certain limitations of the proposed system must be considered. On the one hand, the data segmentation approach used in this work presents some disadvantages when dealing with slow-changing physiological signals, as stated in Section 2.3.1. Different techniques should be applied to take into account all the different physiological particularities without wasting information. For instance, specifically for the GSR, the use of dynamic data segmentation and overlapping could be a valid solution. However, when dealing with resource-constrained devices, a better solution might be to keep track of the onsets of the ERSCRs and, when detecting the offsets for the successive processing windows, calculate all the ERSCRs metrics. The main advantage of this latter method is the independence of the processing window length at the expense of storing the ERSCR tracking information until the completion of the ERSCR (offset). On the other hand, no normalization is applied for the subject-dependent models. Conversely, the z-score is used for the subject-independent model to deal with inter-difference, as stated in Section 5. Despite using these specific normalization strategies, other approaches might be exploited. For instance, the authors are already working on applying different normalization techniques, such as using recovery time-slots to normalize the data of the audiovisual stimulus and study the effect for the analyzed fear use case. At the same time, and following this upgrading context, the authors are also considering some feature selection techniques, such as principal component analysis, sequential feature selection or recursive feature elimination to reduce the redundancy and dimensionality of the problem and to reinforce the system without compromising the model performance. Finally, it should be noted that the convenience of the three initially selected sensors (ECG, GSR, SKT) was validated according to the quality of the assessment metrics presented above. Thus, the results shown are limited by the size of the dataset considered, which is the weakest point of this work. As no other dataset exists that fits the use case, a larger and better dataset is required to create a more reliable system, as stated in Section 5.

Comparing the proposal to related works, the authors checked that the proposed subject-dependent fear detection model outperformed the state-of-the-art approach regarding fear detection. Additionally, other related works within the emotion recognition field were also outperformed. Regarding the subject-independent fear detection model, to the authors’ knowledge, this is the first time that a subject-independent model based on fear detection using a fear binary mapping by means of PAD and discrete emotions and three physiological signals has been presented. This fact is especially valuable due to the advantages of the subject-independent model in comparison to the subject-dependent model.

Some of the limitations identified while performing this work confirm the relevance of creating a novel data set focused on fear detection. This dataset should include some key facts, such as the usage of emotional immersive technology, the modification of the labeling methodology to consider the gender perspective, a properly balanced stimuli distribution regarding the target emotions and a greater number of participants. Thus, as a future work, the authors plan to develop a specific database using professional equipment and our developed hardware platform [56,57], which includes inconspicuous wearable physiological smart sensors. This database will be focused on vulnerable groups, such as gender-based violence victims. Moreover, the labeling process will be done based on a gender perspective, taking into consideration label balancing issues and using virtual reality to produce a stronger emotional immersion. This specific work is currently under development [7]. The resultant intelligent system will be implemented into an embedded wearable device, which will be part of a cyber-physical system that is also connected to trusted responders or even law enforcement agencies.

## Figures and Tables

**Table 1:** Features extracted for the proposed wearable-ready fear recognition system. IBI: inter-beat interval; RP: recurrence plot.

Sensor	Domain	Features
ECG	Time-domain:	Mean of IBI
(25)	(2)	Heart rate variability
	Frequency-domain:	Power spectral density of 4 bands
	(9)	(0–0.1 Hz, 0.1–0.2 Hz, 0.2–0.3 Hz and 0.3–0.4 Hz)
		Power spectral density on IBI for
		Low frequency (LF) (<0.08 Hz)
		Medium frequency (MF) (0.08–0.15 Hz)
		High frequency (HF) (0.15–0.5 Hz)
		Total energy ratio for MF
		Spectral density ratio between
		LF and HF band
	Non-linear:	Multiscale entropy at five levels
	(14)	Detrended fluctuation for raw data
		Detrended fluctuation for IBI
		Recurrence rate
		Determinism
		Laminarity
		Longest RP diagonal line
		Diagonal lines entropy
		Trapping time
		Correlation dimension
GSR	Time-domain:	ER-SRRs including number of peaks,
(17)	(6)	Amplitude and rise time
		Raw data mean value
		Standard deviation
		First quartile
		Third quartile
	Frequency-domain:	Power spectral density of 2 bands
	(3)	for tonic and phasic components
		(0–0.05 Hz, 0.05–1.5 Hz)
		Spectral density ratio for 0–0.05 Hz
	Non-linear:	Detrended fluctuation
	(8)	Recurrence rate
		Determinism
		Laminarity
		Longest RP diagonal line
		Diagonal lines entropy
		Trapping time
		Correlation dimension
SKT	Time-domain:	Raw data mean value
(6)	(4)	Standard deviation
		Skewness
		Kurtosis
	Frequency-domain:	Power spectral density of two bands
	(2)	(0–0.1 Hz, 0.1–0.2 Hz)

**Table 2:** Performance metrics for each generated subject-dependent model and average performance metrics and dispersion for each classification algorithm. SVM: support vector machine; KNN: k-nearest neighbor; ENS: ensemble classifiers; MAD: mean absolute deviation; ACC: accuracy; AUC: area under the curve.

			SVM				KNN				ENS		
Training	Trained	ACC	AUC	Gmean	F1	ACC	AUC	Gmean	F1	ACC	AUC	Gmean	F1
Type	Volunteers	(MAD)	(MAD)	(MAD)	(MAD)	(MAD)	(MAD)	(MAD)	(MAD)	(MAD)	(MAD)	(MAD)	(MAD)
Subject dependent	V1	89.00%	90.30%	87.73%	85.71%	88.00%	88.67%	87.90%	85.37%	88.00%	79.32%	85.12%	83.33%
	V2	88.00%	92.43%	76.41%	71.43%	99.00%	99.89%	99.23%	87.72%	91.00%	97.47%	85.41%	80.85%
	V3	91.00%	94.44%	71.20%	74.29%	94.00%	96.19%	90.47%	85.00%	97.00%	95.31%	98.13%	93.02%
	V4	93.00%	95.29%	84.06%	75.86%	99.00%	96.67%	96.59%	96.55%	96.00%	99.69%	85.62%	84.62%
	V5	76.00%	84.97%	75.01%	72.09%	81.00%	91.47%	85.62%	84.62%	98.00%	99.88%	97.95%	97.78%
	V6	90.00%	93.67%	87.92%	83.33%	98.00%	98.60%	84.08%	82.86%	99.00%	99.90%	99.23%	98.36%
	V7	93.00%	98.54%	92.47%	91.14%	100.00%	100.00%	100.00%	100.00%	100.00%	100.00%	100.00%	100.00%
	V8	85.00%	90.57%	81.22%	74.58%	94.00%	92.24%	90.58%	89.29%	93.00%	86.05%	92.12%	88.52%
	V9	96.00%	98.44%	83.16%	77.78%	99.00%	99.44%	99.40%	95.24%	100.00%	100.00%	100.00%	100.00%
	V10	89.00%	91.31%	87.73%	85.71%	94.00%	94.15%	93.24%	92.31%	100.00%	100.00%	100.00%	100.00%
	V11	95.00%	50.00%	00.00%	00.00%	99.00%	90.00%	89.44%	88.89%	100.00%	100.00%	100.00%	100.00%
	V12	77.00%	83.48%	62.91%	53.06%	91.00%	85.95%	84.97%	83.02%	94.00%	93.33%	90.58%	89.29%
		88.50%	88.62%	74.15%	70.42%	94.66%	94.44%	91.80%	89.24%	96.33%	95.91%	95.51%	92.98%
		(4.66%)	(7.90%)	(14.72%)	14.62%	(4.33%)	(4.02%)	(4.92%)	(4.53%)	(3.28%)	(4.94%)	(5.62%)	(6.38%)

**Table 3:** Performance metrics for each generated subject-independent model and average performance metrics and dispersion for each classification algorithm. The training stage is performed using all the volunteers except the tested volunteer in each model generated (unseen test data).

			SVM				KNN				ENS		
Training	Tested	ACC	AUC	Gmean	F1	ACC	AUC	Gmean	F1	ACC	AUC	Gmean	F1
Type	Volunteers	(MAD)	(MAD)	(MAD)	(MAD)	(MAD)	(MAD)	(MAD)	(MAD)	(MAD)	(MAD)	(MAD)	(MAD)
Subject independent	V1	65.00%	60.83%	57.15%	47.76%	75.00%	71.25%	68.74%	62.69%	71.00%	65.83%	60.55%	52.46%
	V2	70.00%	61.33%	58.83%	42.31%	81.00%	74.00%	72.66%	61.22%	82.00%	86.72%	71.26%	60.87%
	V3	64.00%	66.00%	62.44%	40.00%	72.00%	61.88%	59.53%	39.13%	62.00%	61.19%	45.82%	24.00%
	V4	82.00%	71.01%	83.88%	59.09%	84.00%	87.84%	87.67%	63.64%	85.00%	91.61%	90.75%	66.67%
	V5	64.00%	70.55%	61.10%	55.00%	70.00%	71.74%	65.32%	59.46%	73.00%	75.58%	68.16%	63.01%
	V6	84.00%	88.57%	85.61%	77.14%	71.00%	68.81%	68.59%	56.72%	79.00%	87.86%	76.16%	66.67%
	V7	75.00%	90.54%	65.38%	59.02%	76.00%	91.83%	69.37%	63.63%	87.00%	99.46%	82.16%	80.60%
	V8	76.00%	81.90%	70.51%	60.00%	78.00%	72.86%	71.71%	62.07%	80.00%	85.00%	75.59%	66.67%
	V9	67.00%	69.67%	63.77%	21.82%	67.00%	59.44%	58.69%	18.87%	78.00%	84.78%	78.88%	42.11%
	V10	76.00%	79.63%	65.95%	60.00%	78.00%	72.92%	68.34%	63.33%	77.00%	82.30%	72.80%	67.61%
	V11	74.00%	90.53%	76.78%	23.53%	80.00%	89.47%	88.85%	40.00%	74.00%	86.32%	85.22%	27.78%
	V12	70.00%	72.05%	64.14%	51.61%	71.00%	66.90%	66.12%	53.97%	72.00%	67.72%	66.73%	54.84%
		72.25%	75.22%	67.96%	49.77%	75.25%	74.07%	70.47%	53.73%	76.67%	81.20%	72.84%	56.11%
		(5.58)	(9.18)	(7.48)	(12.24)	(4.25)	(7.82)	(6.50)	(10.53)	(5.22)	(9.07)	(8.62)	(13.22)

**Figure A1:**
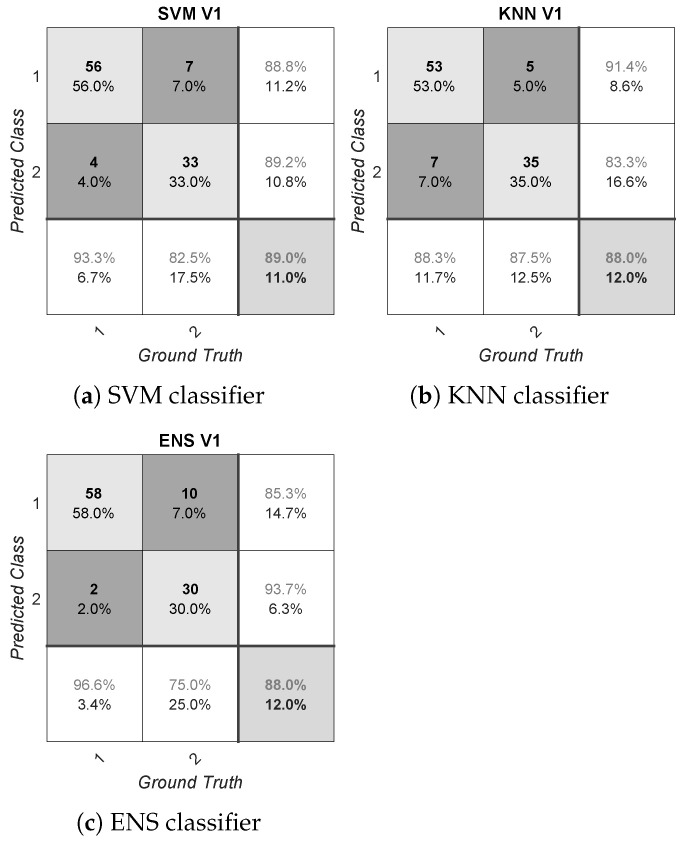
Confusion matrices for a subject-dependent model for V1.

**Figure A2:**
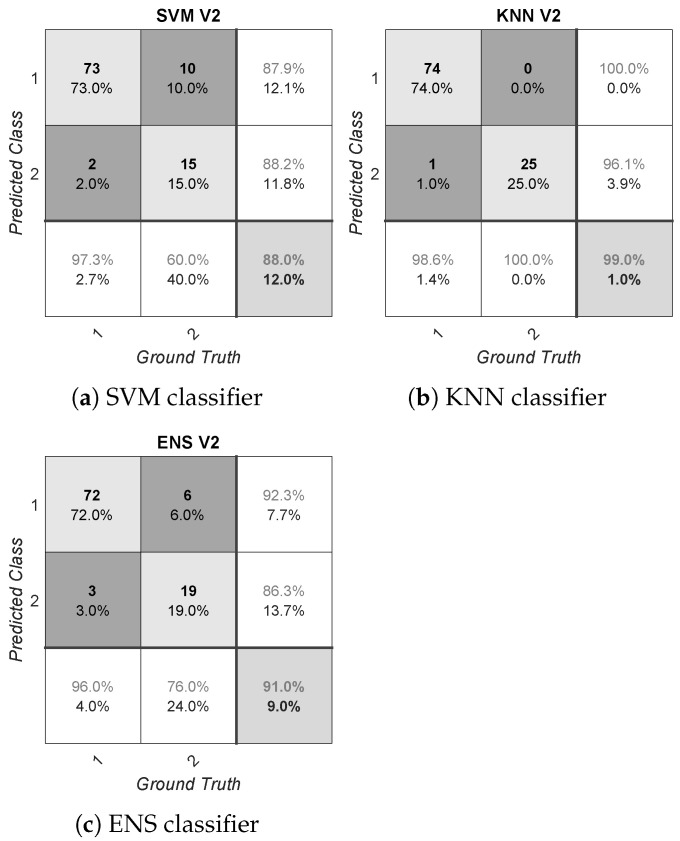
Confusion matrices for a subject-dependent model for V2.

**Figure A3:**
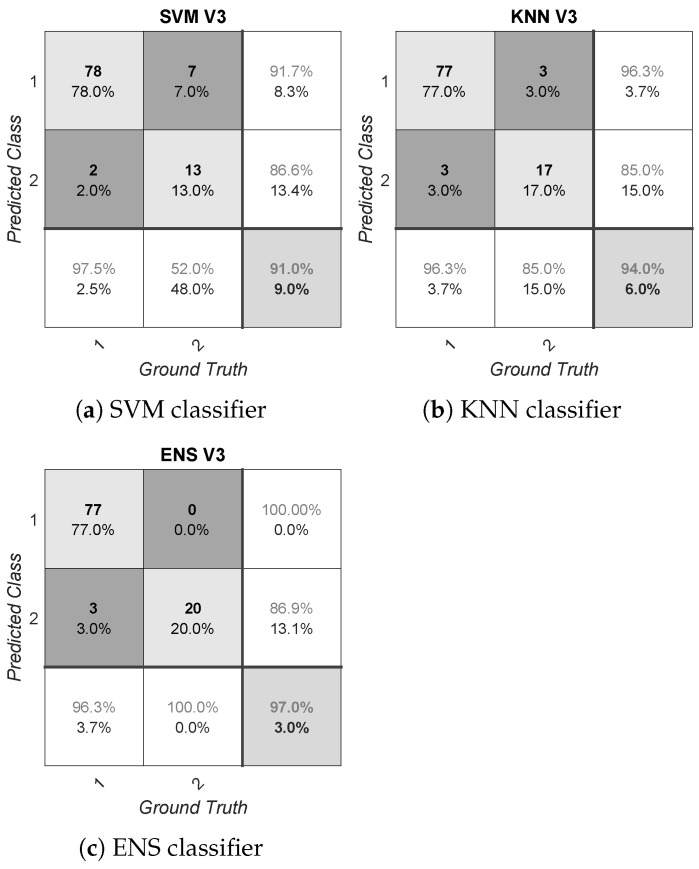
Confusion matrices for a subject-dependent model for V3.

**Figure A4:**
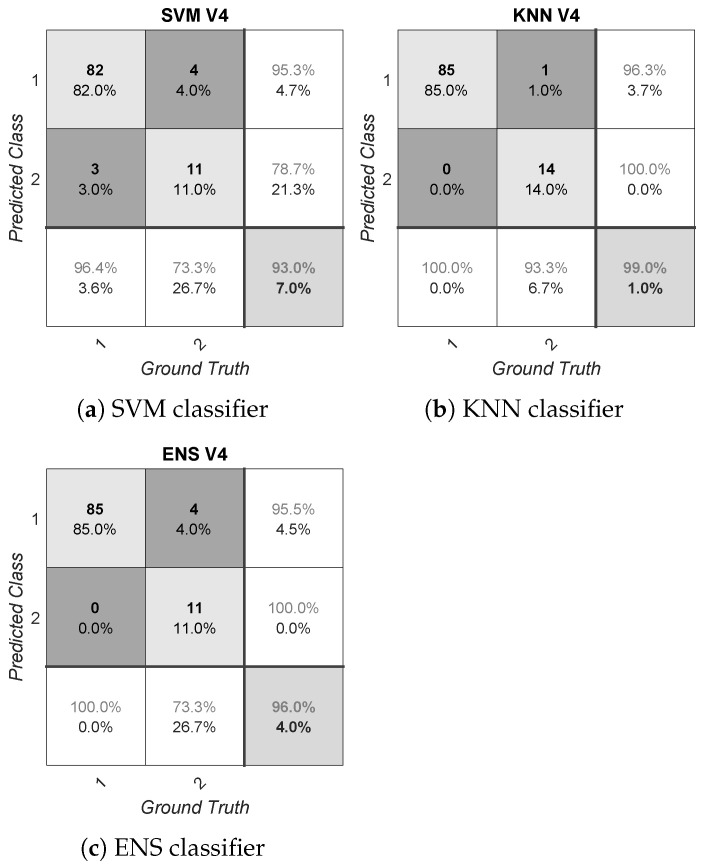
Confusion matrices for a subject-dependent model for V4.

**Figure A5:**
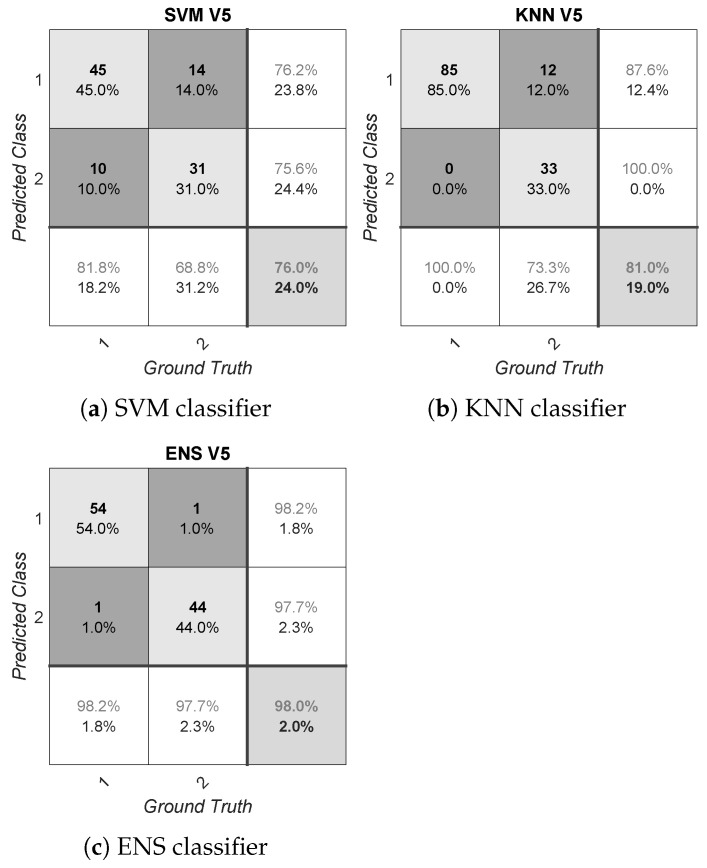
Confusion matrices for a subject-dependent model for V5.

**Figure A6:**
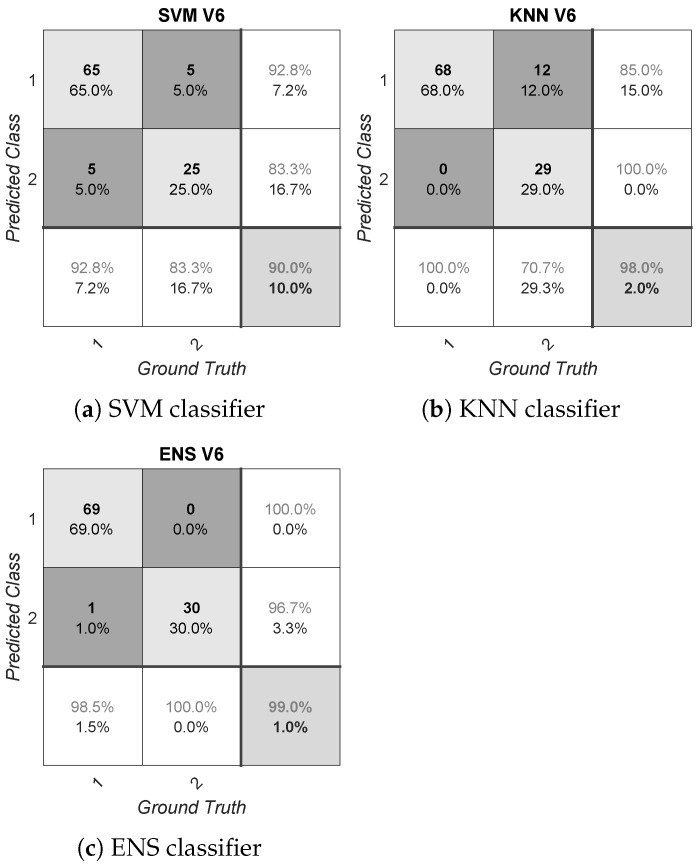
Confusion matrices for a subject-dependent model for V6.

**Figure A7:**
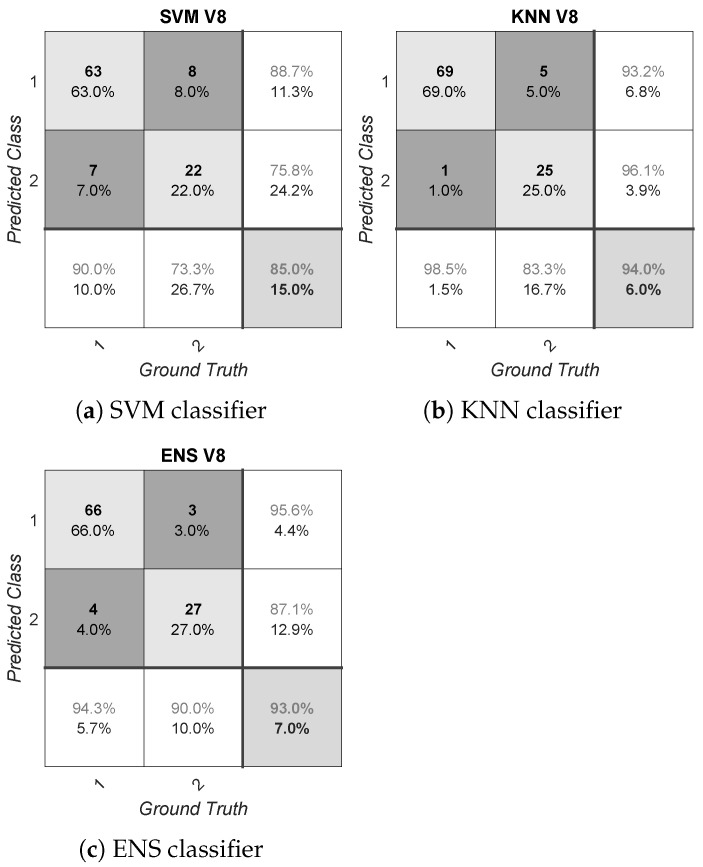
Confusion matrices for a subject-dependent model for V8.

**Figure A8:**
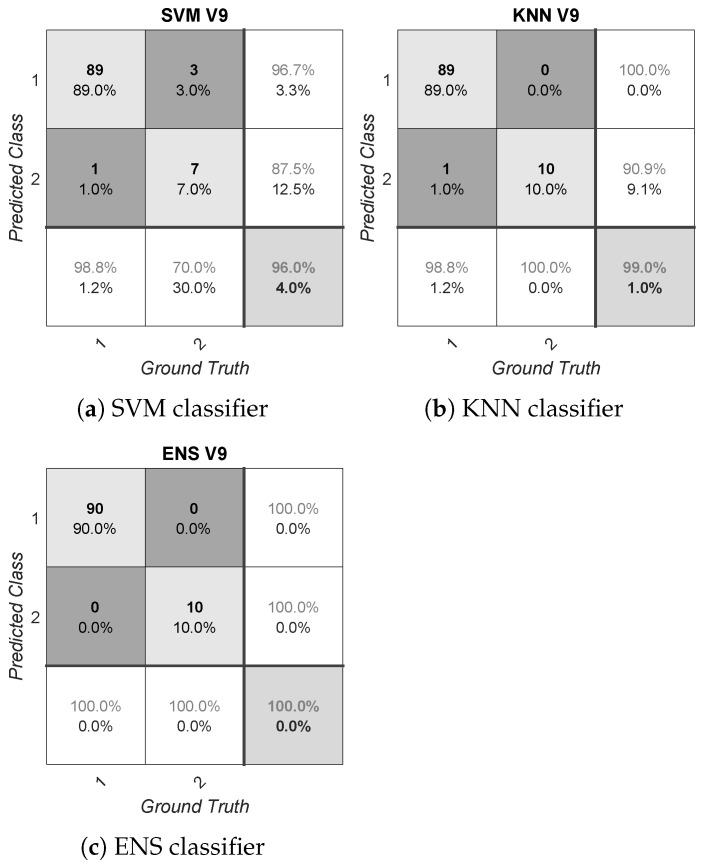
Confusion matrices for a subject-dependent model for V9.

**Figure A9:**
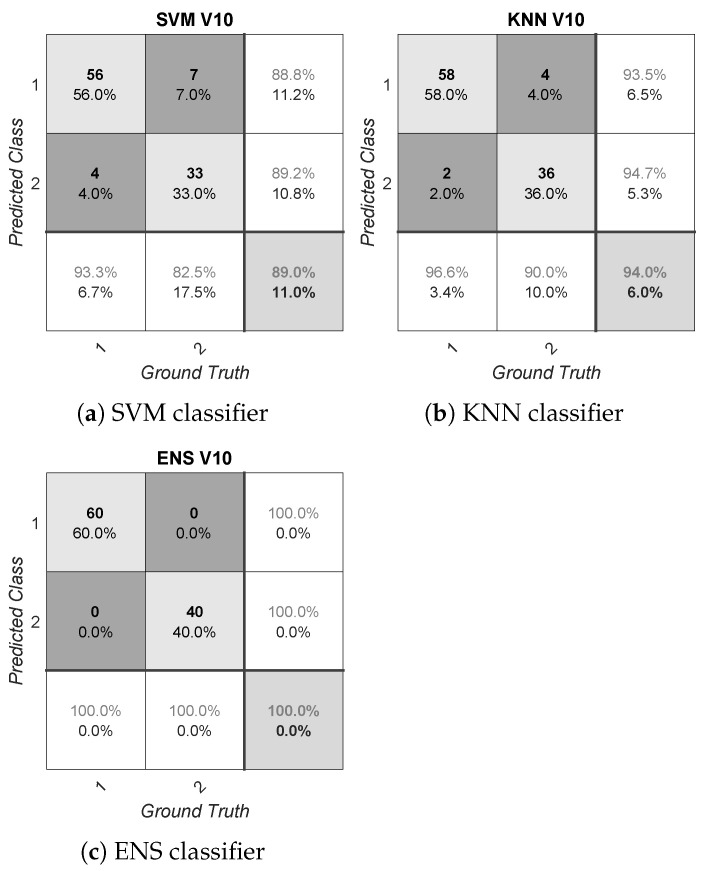
Confusion matrices for a subject-dependent model for V10.

**Figure A10:**
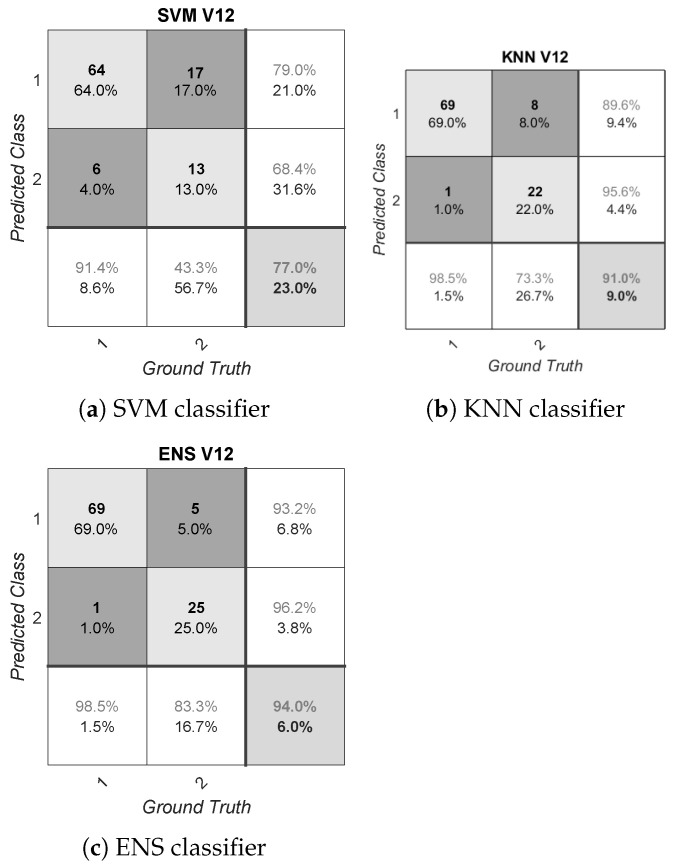
Confusion matrices for a subject-dependent model for V12.

**Figure A11:**
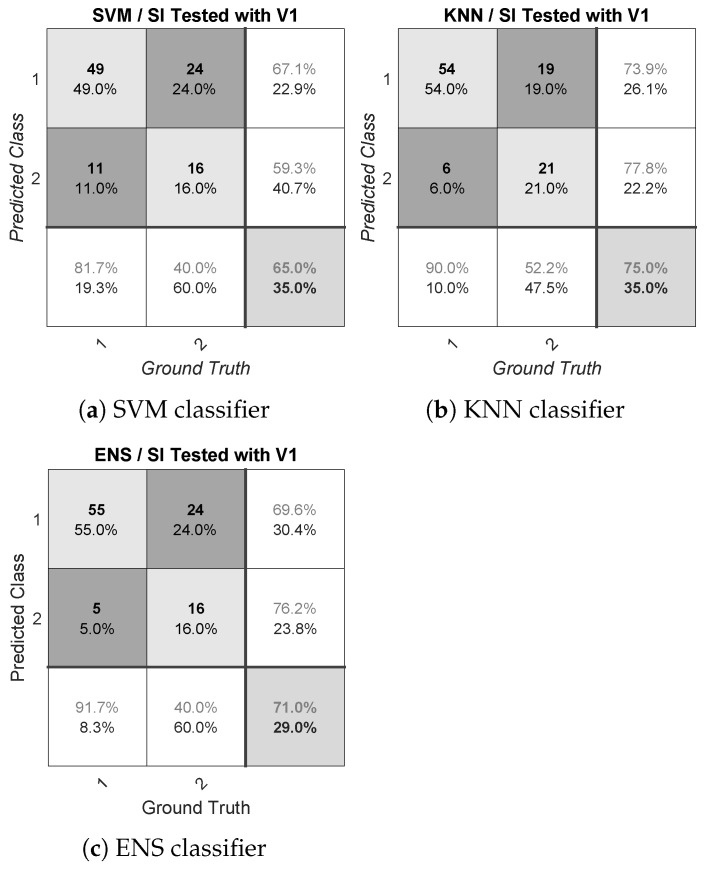
Confusion matrices for a subject-independent model tested with V1.

**Figure A12:**
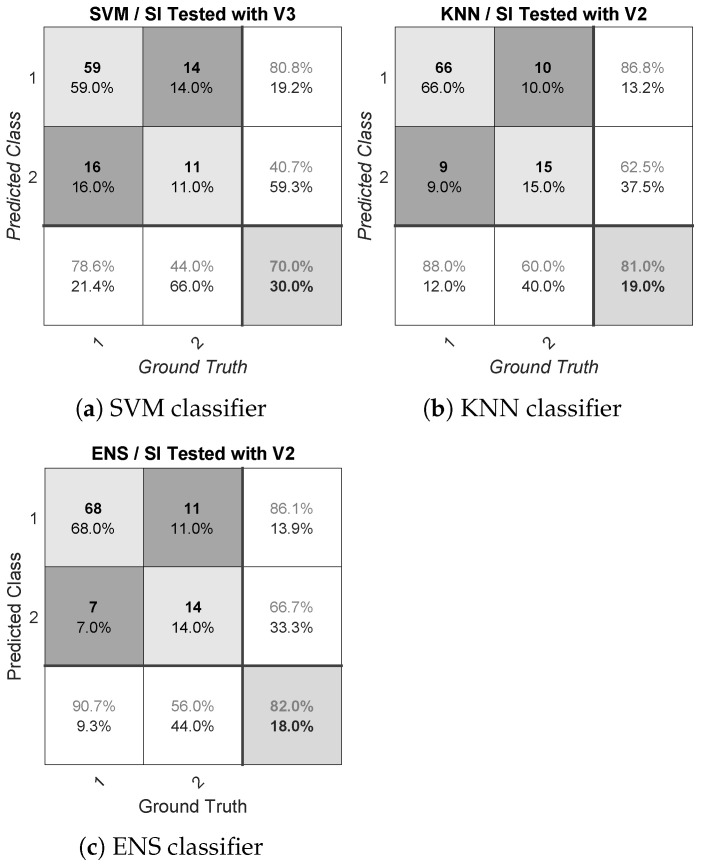
Confusion matrices for a subject-independent model tested with V2.

**Figure A13:**
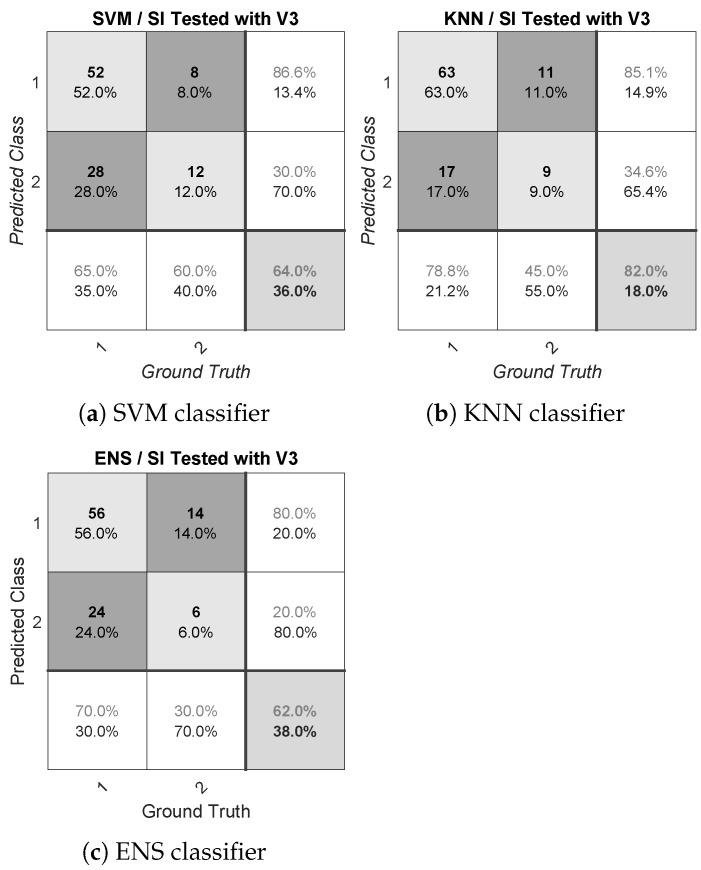
Confusion matrices for a subject-independent model tested with V3.

**Figure A14:**
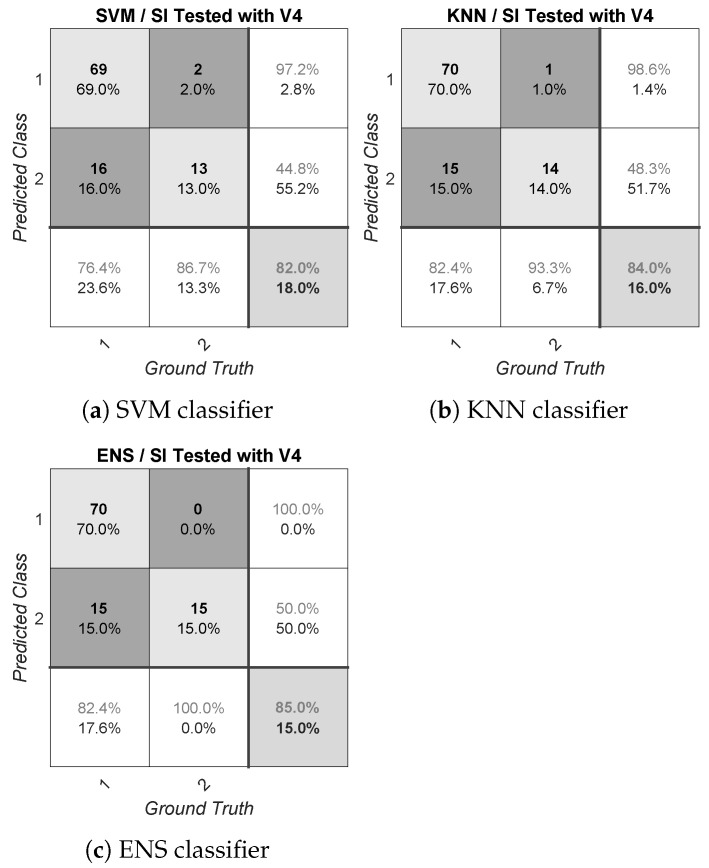
Confusion matrices for a subject-independent model tested with V4.

**Figure A15:**
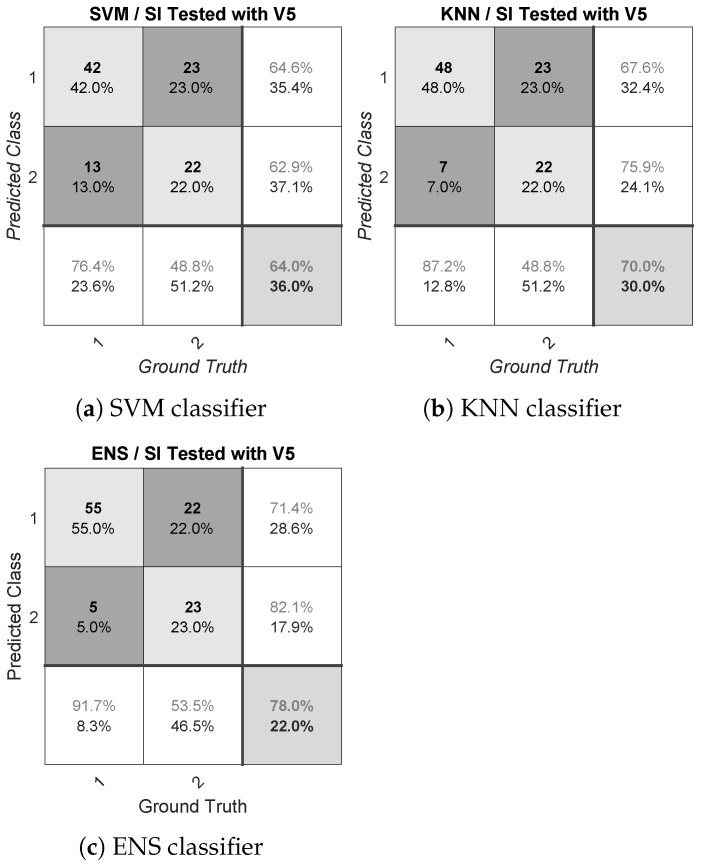
Confusion matrices for a subject-independent model tested with V5.

**Figure A16:**
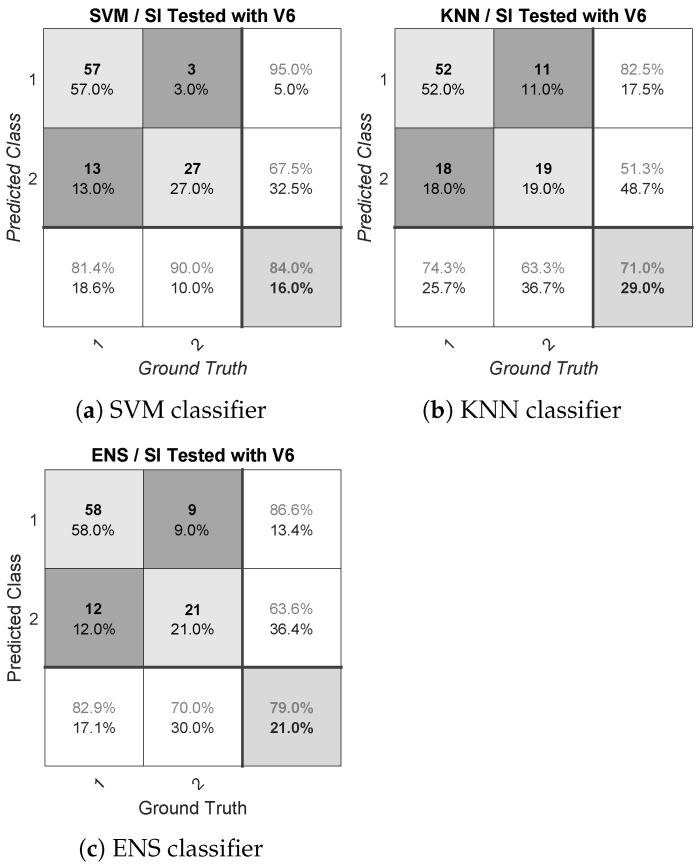
Confusion matrices for a subject-independent model tested with V6.

**Figure A17:**
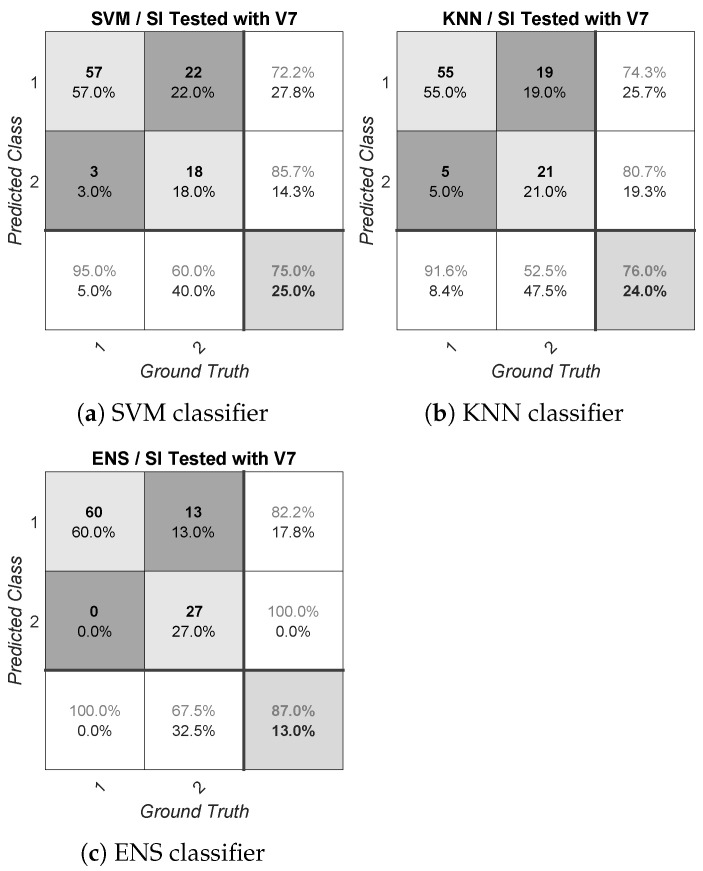
Confusion matrices for a subject-independent model tested with V7.

**Figure A18:**
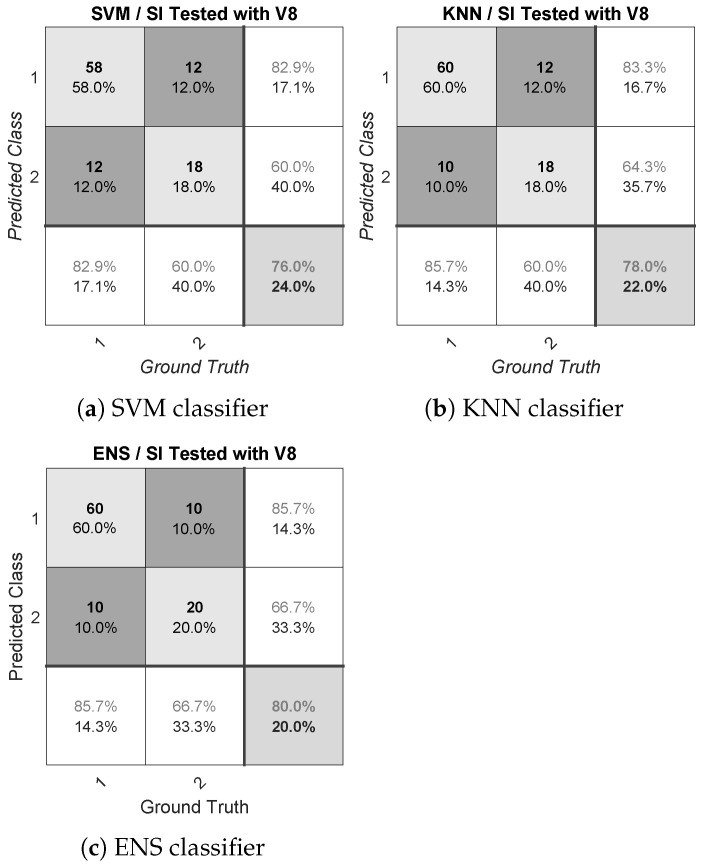
Confusion matrices for a subject-independent model tested with V8.

**Figure A19:**
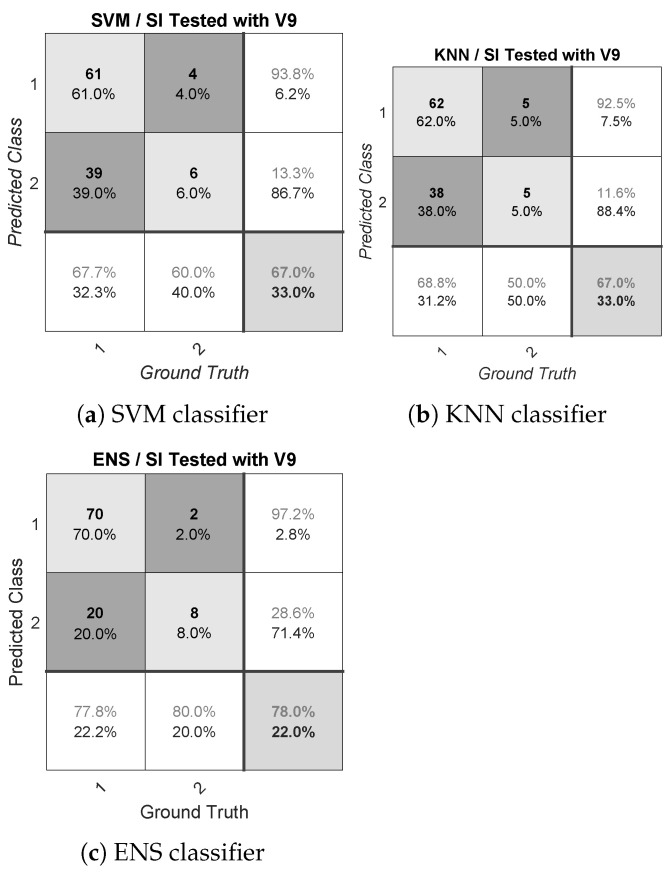
Confusion matrices for a subject-independent model tested with V9.

**Figure A20:**
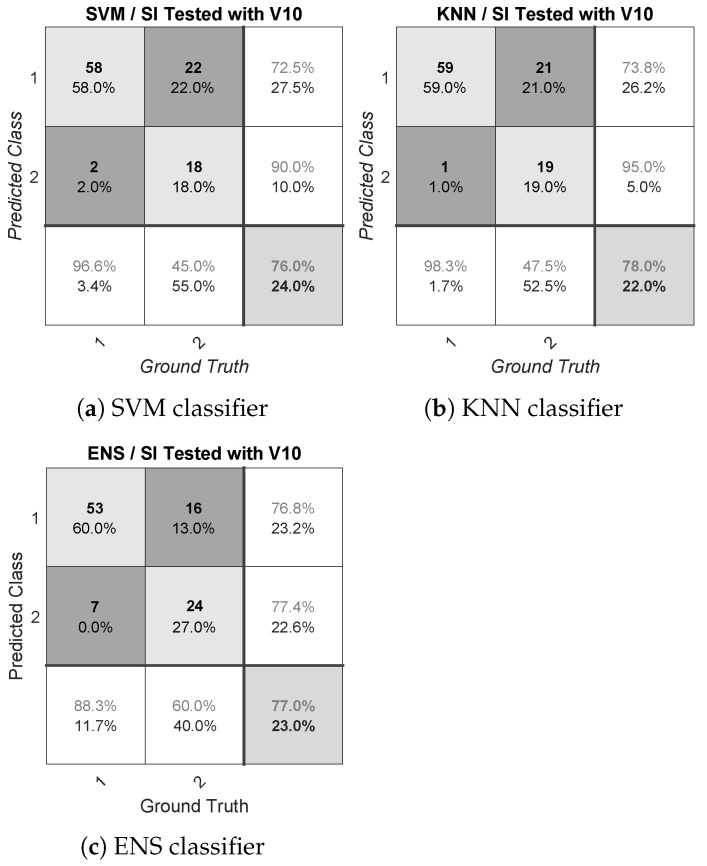
Confusion matrices for a subject-independent model tested with V10.

**Figure A21:**
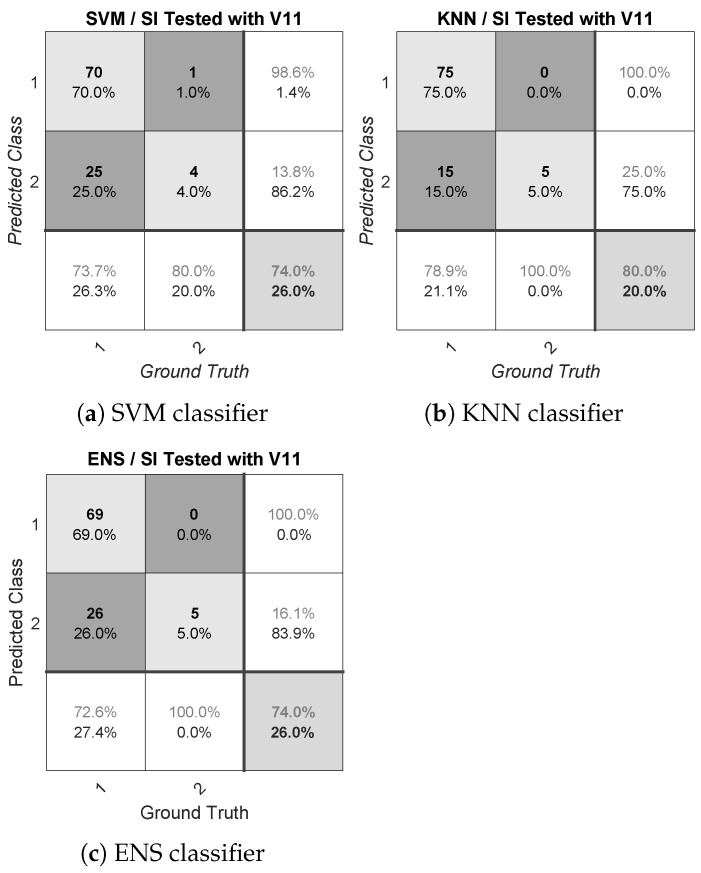
Confusion matrices for a subject-independent model tested with V11.

**Figure A22:**
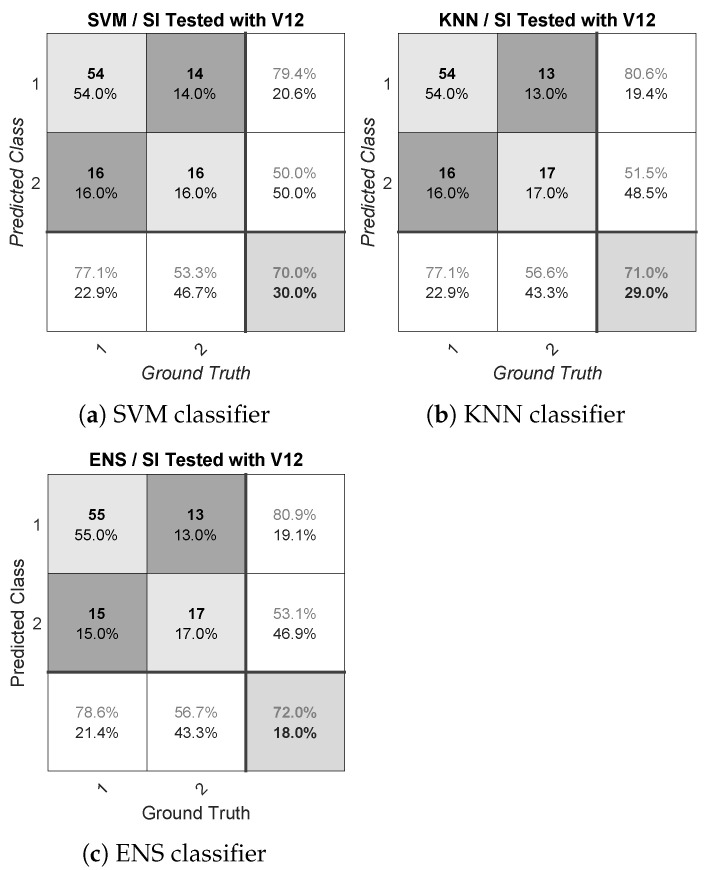
Confusion matrices for a subject-independent model tested with V12.

## Data Availability

Not applicable.
